# A retrospective analysis of the management of renal hyperparathyroidism; evaluating changes in practice and outcome in an era of calcimimetics

**DOI:** 10.1007/s00423-025-03744-2

**Published:** 2025-06-02

**Authors:** William P. Duggan, Rory Patterson, Niamh M. Smyth, Niamh Kyne, David Synnott, Nathaniel McHugh, Ruey Ying Teo, Rhodri Hill, Umar Khan, Sharjeel Paul, Donal Reddan, Catherine Wall, William Plant, John Kinsella, Orla Young, Aoife Lowery, Paul Redmond, Peter Conlon, Arnold D. K. Hill

**Affiliations:** 1 Department of Surgery, Royal College of Surgeons in Ireland, Dublin 9, Ireland; 2 Department of Kidney Transplantation, Beaumont Hospital, Dublin 9, Ireland; 3 Department of Ear, Nose and Throat Surgery, University Hospital Galway, Galway, Ireland; 4 Department of Surgery, University Hospital Galway, Galway, Ireland; 5 Department of Surgery, Cork University Hospital, Cork, Ireland; 6 Department of Ear, Nose and Throat Surgery, Tallaght University Hospital, Dublin, Ireland; 7 Department of Nephrology, University Hospital Galway, Galway, Ireland; 8 Department of Nephrology, Tallaght University Hospital, Dublin, Ireland; 9 Department of Nephrology, Cork University Hospital, Cork, Ireland; 10 Beaumont Hospital, Beaumont Road, Dublin 9, Ireland

**Keywords:** Parathyroid surgery, Renal hyperparathyroidism, Calcimimetics

## Abstract

**Purpose:**

Hyperparathyroidism (HPT) is a common and significant complication of chronic kidney disease (CKD). Both parathyroidectomy and cinacalcet, are used routinely in an effort to manage this cohort. Unfortunately, there remains no guideline consensus on how best to combine these treatments into an effective strategy**.** We look to assess the efficacy of these interventions and identify factors predicting recurrence and the development of post-operative complications. We also examine changes in our practice nationally following the arrival of cinacalcet as an alternative or an abridge to definitive surgical management.

**Methods:**

This was a nationwide study. We conducted a retrospective analysis of a prospectively maintained database. All patients who underwent a parathyroidectomy as management of secondary or tertiary HPT between 1999 and 2023 were included. A control group of patients managed with cinacalcet were also included.

**Results:**

Our cohort included 155 patients managed with parathyroidectomy and 203 patients treated with cinacalcet. Pre-operative Alkaline phosphatase > 200 IU/L was predictive of hungry bone syndrome (HBS) on univariate (*P* = 0.003) and multivariate (*P* = 0.002) analysis, whilst a PTH > 1000 pg/ml (*P* = 0.012) was also predictive of HBS on univariate analysis. In an attempt to identify an optimal PTH cut off to trigger surgical referral we found mean serum PTH levels were significantly higher at 5 years in the cohort of patients who had a PTH > 1000 pg/ml prior to surgical intervention (39 ± 32 Vs 374 ± 544, *P* = 0.045).

**Conclusions:**

Our findings re-emphasise the efficacy and safety of parathyroid surgery in the management of renal HPT and suggest earlier surgical referral may improve the incidence of post-operative HBS and recurrent HPT.

**Supplementary Information:**

The online version contains supplementary material available at 10.1007/s00423-025-03744-2.

## Introduction

Chronic kidney disease is a common condition that affects 10–15% of the world’s population [1, 2]. Renal hyperparathyroidism (HPT) remains a significant complication of chronic kidney disease, often requiring intervention to manage its effects on calcium-phosphate homeostasis and bone metabolism [3]. Renal HPT is classically broken into 2 types. Secondary HPT is the elevation of parathyroid hormone (PTH) in response to hypocalcaemia induced by phosphate retention and reduced calcitriol synthesis as a consequence of reduced renal function [4]. In secondary disease, all the parathyroid glands become enlarged owing to parathyroid hyperplasia. Because secondary hyperplasia is a compensatory mechanism, it commonly resolves following normalization of calcium and phosphorus homeostasis resulting from management of the underlying kidney dysfunction. Tertiary HPT is seen when a patient with longstanding secondary disease develops autonomous PTH secretion. This is observed in up to 30% of patients with end stage renal disease who have undergone a renal transplant [5].

Surgical parathyroidectomy, traditionally considered the definitive treatment, has seen changes in practice with the advent of pharmacological alternatives such as calcimimetics, particularly cinacalcet. Cinacalcet is a calcimimetic agent that acts by activating calcium-sensing receptors in the parathyroid gland to lower serum PTH levels [6]. Many randomised control trials have demonstrated that cinacalcet is superior to more conventional pharmacological approaches in achieving treatment targets in renal HPT [6, 7]. Cinacalcet is however also associated with a significant side –effect profile leading to issues with compliance, it is also a considerable financial burden particularly for patients requiring treatment for prolonged durations [8, 9].

Despite the emergence of novel treatment alternatives, there remains no guideline consensus on how best to combine these treatments into an effective strategy [10–12]. This study evaluates the evolving landscape of renal HPT management by comparing surgical outcomes with those of medical management using cinacalcet. Utilising a national collaborative dataset from five renal centres, we look to assess the efficacy of these interventions and identify factors predicting recurrence and the development of post-operative complications, such as hungry bone syndrome (HBS)*.* We also examine changes in our practice nationally following the arrival of cinacalcet as an alternative or an abridge to definitive surgical management.

## Methods

### Study population

The study population included a series of patients who underwent surgical management of renal HPT across 5 national renal centres between January 1 st, 1999 and December 31 st, 2023, as identified from prospectively maintained institutional registries (See supplementary Table 1). A retrospective review of electronic records was performed to analyse the relevant demographics and biochemical data. A chart review was performed to identify missing data not recorded electronically. A control cohort of patients from The National centre for Renal transplantation at Beaumont Hospital managed with cinacalcet between 2009 and 2023 were also included for the purpose of comparative analysis.

### Surgical technique

Our entire surgical cohort underwent either a 3.5 gland subtotal parathyroidectomy (SPTX) or a total parathyroidectomy with auto-transplantation (TPTX + AT). SPTX technique; following clear identification of all 4 parathyroid glands, 3 of the glands are fully excised, whilst a partial resection of the final gland is performed leaving behind a well vascularised remnant the size of a normal parathyroid. TPTX + AT technique; all four glands are clearly identified and removed, one parathyroid gland is divided and re-implanted into muscle in the forearm. A number of meta- analyses have previously been performed to compare outcomes amongst these two surgical approaches. They have found no significant difference between the approaches with regard to initial success, recurrence, reoperation rate, or long-term hypocalcaemia [13, 14]. Patients who had no follow-up beyond their surgical admission were excluded. Surgical approach was based on individual surgeon preference, not patient factors, therefore we do not anticipate any selection bias.

### Data collection

The following data was collected for each patient in the surgical cohort; age, sex, procedure type, whether the patient received a successful kidney transplant, whether the patient was treated with cinacalcet, if the patient was symptomatic prior to parathyroidectomy. Data was collected regarding patients who had high PTH or calcium levels post parathyroid surgery. For the purpose of analysis, patients who had post-operative parathyroid imaging in the form of an ultrasound or a technetium sestamibi scan were categorised as having recurrent disease. HBS is defined as a severe drop in serum total calcium concentration less than 2.1 mmol/L and/or prolonged hypocalcaemia for more than 4 days [15]. The nature of this study prevented accurate evaluation of these parameters retrospectively, so by proxy we have categorised any patients who received intravenous calcium supplementation post-operatively as having HBS. This was felt to be an appropriate step given, intravenous calcium is generally reserved only for patients with significant post-operative hypocalcaemia. Data was collected regarding post-operative complications including graft dysfunction. Psychiatric complications relating to hypercalcaemia included; depression, anxiety and psychosis. Symptomatic bone disease included patients suffering with refractory bone pain or patients who developed pathological fractures secondary to hypercalcaemia. The following data was collected for each patient in the cinacalcet cohort; sex, duration of treatment, maximum dosage and reasons beyond kidney transplantation or parathyroid surgery and why cinacalcet was discontinued. As cinacalcet was introduced into mainstream practice in Ireland in 2009, the pre-cinacalcet era as mentioned in the manuscript includes patients who underwent parathyroid surgery between 1999 and 2008, whilst the post-cinacalcet era describes patients who underwent surgery between 2009 and 2023.

### Statistical analysis

Continuous variables are mean ± standard deviation, categorical variables are n (%). The distribution of continuous variables was compared between groups using a Mann–Whitney U test for unpaired non-parametric variables and an unpaired t-test for parametric variables. Categorical variable frequency was compared between groups using the Chi square test. A *P*-value of 0.05 was defined as the cut-off for statistical significance. Univariate and multivariate linear regression analysis was performed using PTH decrease, incidence of recurrence (this included patients who had imaging performed to identify a source of recurrence) and HBS as the dependant variable in each instance. All statistical analyses were performed using STATA 12.1 (StataCorp, College Station, TX).

## Results

### Clinical characteristics and demographics

#### Parathyroidectomy cohort

155 parathyroidectomies were performed in the context of renal HPT between 1999 and 2023 (See Table 1). 69.7% of patients underwent a 3.5 gland SPTX, with the remaining 30.3% undergoing TPTX + AT. 69.1% of patients were in receipt of a kidney transplant prior to undergoing parathyroid surgery. 54% of patients were on cinacalcet prior to referral for definitive parathyroid surgery. 27% of patients were symptomatic pre-operatively, symptomatic bone disease (18.7%) was the most commonly documented complication. 27.7% of patients developed HBS post-operatively. 19.3% of patients were re-imaged post parathyroidectomy to investigate for recurrent disease, however only 2.6% of patients required further surgical intervention as part of any management of suspected recurrence. Other post-operative complications were encountered less commonly. Importantly there were only 2 documented instances of graft dysfunction post parathyroidectomy.

**Table 1:** Patient, clinical and operative characteristics of our parathyroidectomy cohort. Continuous variables are mean ± standard deviation, categorical variables are n (%). Symptomatic from a bone perspective includes both patients with bone pain or fragility fractures. ‘*Hungry bone syndrome’* was defined as any patient who required IV calcium supplementation or developed symptomatic hypocalcaemia post parathyroidectomy*. PTX parathyroidectomy, TPTX* + *AT total parathyroidectomy and autotransplantation, US ultrasound, LBO large bowel obstruction*

	Parathyroidectomy (*n* = 155)
Age	49 ± 15
Male	77(**49.7%**)
Surgical Approach	
3.5 gland PTX	108 (**69.7%**)
TPTX + AT	47 (**30.3%)**
Transplanted before PTX	107(**69.1%**)
On cinacalcet before PTX	50/91 **(54.9%)**
Symptomatic Pre-operatively	42 (**27.1%**)
Bone	29 **(18.7%)**
Psychiatric	11 (**7.1%**)
Renal Calculi	6 **(3.8%)**
Pseudogout	3 **(1.9%)**
Recurrence	
Underwent Post-operative imaging (Sestamibi/US)	30(**19.3%**)
Required further surgical intervention	4(**2.6%**)
*Hungry Bone Syndrome*	43(**27.7%**)
Post-operative graft dysfunction	2/108 (**1.9%**)
Other peri-operative complication	
Haematoma (requiring return to theatre for evacuation)	3(**1.9%**)
Hoarseness	2(**1.2%**)
Post-op pneumonia	2(**1.2%**)
LBO requiring laparotomy	1(**0.6%**)
Post-operative hyponatraemia	2 (**1.2**%)

#### Cinacalcet cohort

We included 203 consecutive patients who had been commenced on cinacalcet between 2009 and 2023 in the context of renal HPT, as a control group to allow comparative analysis (See Table 2). 19 patients from our cinacalcet control group eventually required parathyroid surgery and are therefor also represented in the parathyroid surgery group. 48.7% of patients were in receipt of a kidney transplant prior to being commenced on cinacalcet. The minimum recorded maximum daily dose was 15 mg (1.9%), with one patient reaching a maximum daily dose of 180 mg. 60.6% of patients reached a maximum once daily dose of 30 mg. The mean duration of treatment was 31 months, with 35.9% of patients discontinuing treatment for reasons other than kidney transplantation or referral for definitive parathyroid surgery. Gastro-intestinal (GI) disturbance was the most commonly documented alternative reason for discontinuation of treatment, though it should be mentioned that following review of relevant patient records, the specific reason for discontinuing cinacalcet was unclear amongst 58 patients across our cohort.

**Table 2:** Clinical and treatment characteristics of our cinacalcet cohort. Continuous variables are mean ± standard deviation, categorical variables are n (%). *PTX parathyroidectomy*

	Cinacalcet (*n* = 203)
Male	118 **(58.1%)**
Transplanted before commencement of Cinacalcet	99 **(48.7%)**
Max Recorded Dose	
15 mg	4 **(1.9%)**
30 mg	123 **(60.6%)**
60 mg	54 **(26.6%)**
90 mg	14 **(6.9%)**
120 mg	3 **(1.5%)**
180 mg	1 **(0.5%)**
Duration of Treatment	
Months	**31** ± 27
Discontinued for reasons other than transplant or PTX	73 **(35.9%)**
GI Disturbance	10 **(4.9%)**
Hypocalcaemia	4 **(2.0%)**
Hepatitis	1 **(0.5%)**

### Comparing efficacy of interventions in reducing serum PTH and Calcium

**Fig. 1:**
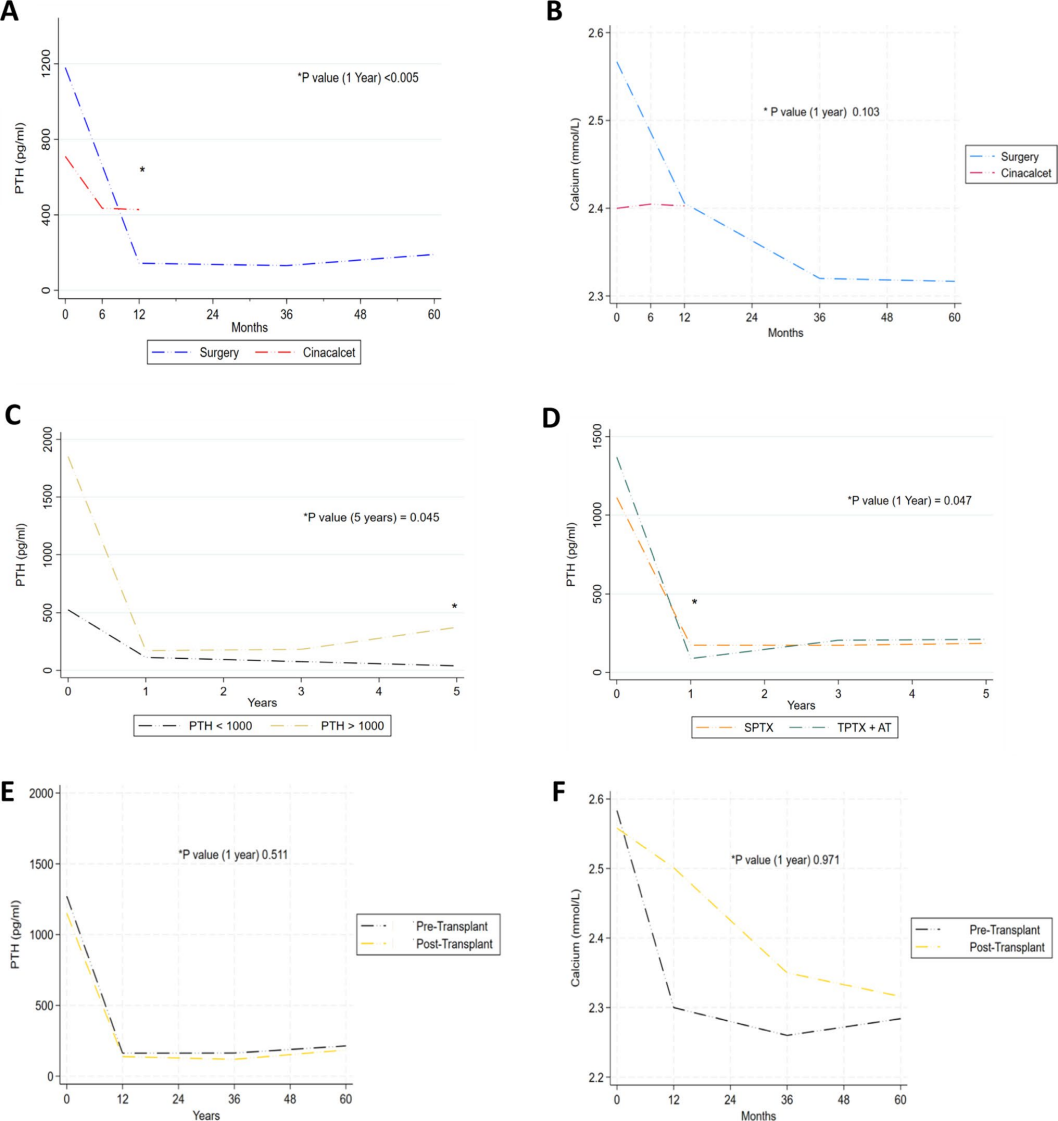
**A** Line graph demonstrating trends in mean serum PTH post surgical intervention or commencement of cinacalcet. **B** Line graph demonstrating trends in mean serum Calcium post surgical intervention or commencement of cinacalcet. **C** Line graph demonstrating trends in mean serum PTH according to whether the serum PTH was above or below 1000 pg/ml in advance of surgical referral. **D** Line graph demonstrating trends in mean serum PTH post either subtotal 3.5 gland parathyroidectomy or total parathyroidectomy with autotransplantation. **E** Line graph demonstrating trends in mean serum PTH in patients who underwent surgical parathyroidectomy either in advance or post receipt of a kidney transplant. **F** Line graph demonstrating trends in mean serum calcium in patients who underwent surgical parathyroidectomy either in advance or post receipt of a kidney transplant. Mann–Whitney U test was used for unpaired non-parametric variables and an unpaired t-test for parametric variables. *PTX parathyroidectomy, SPTX subtotal parathyroidectomy, TPTX* + *AT total parathyroidectomy and autotransplantation*

We looked to establish how different interventions impacted serum PTH and calcium levels at selected intervals. Firstly, we looked to compare surgical intervention with cinacalcet. The mean PTH prior to commencing cinacalcet was 710 pg/ml across our cohort, compared to 1188 pg/ml ahead of referral for parathyroidectomy (See Table 3, Fig. 1A). Meanwhile the mean serum calcium prior to commencing cinacalcet was 2.40 mmol/l, compared to 2.57 mmol/l ahead of surgical referral (See Table 3, Fig. 1B). As anticipated, parathyroidectomy resulted in a significantly greater decrease in PTH at one year as compared to cinacalcet (428 ± 446 Vs 144 ± 246, *P* < 0.005). When comparing surgical approaches, APTX + AT resulted in a statistically more significant mean reduction in PTH at 1 year compared to SPTX (174 ± 222 Vs 88 ± 143, *P* = 0.047), however there were no significant differences in the PTH levels according to surgical approach at 3 or 5 years (See Fig. 1D).

**Table 3:** Comparing the impact of various interventions on reducing serum PTH levels pre-intervention and at 1, 3 and 5 years. Continuous variables are represented as mean ± standard deviation. *PTX parathyroidectomy, SPTX subtotal parathyroidectomy, TPTX* + *AT total parathyroidectomy and autotransplantation*

PTH (pg/ml)	Pre- Intervention	1 Year	3 Years	5 years
Cinacalcet (*n* = 203)	**710** ± 635	**428** ± 446	-	**-**
PTX (All) (*n* = 155)	**1188** ± 934	**144** ± 246	**130** ± 335	**193** ± 399
SPTX (*n* = 108)	**1111** ± 891	**174** ± 222	**173** ± 246	**185** ± 396
TPTX + AT (*n* = 47)	**1369** ± 1015	**88** ± 143	**205** ± 410	**211** ± 405
PTH < 1000 (*n* = 85)	**526** ± 574	**112** ± 158	**76** ± 187	**39** ± 32
PTH ≥ 1000 (*n* = 70)	**1851** ± 574	**171** ± 232	**183** ± 450	**374** ± 544

Next we looked to identify whether there were significant differences in serum PTH levels over time, depending on whether or not the PTH was > 1000pg/ml in advance of surgical intervention (See Fig. 1C). Though we did not identify any difference in serum PTH levels between the groups at 1 or 3 years, mean PTH levels were significantly higher at 5 years in the cohort of patients who had a PTH > 1000 pg/ml prior to surgical intervention (39 ± 32 Vs 374 ± 544, *P* = 0.045).

We also compared changes in serum PTH (See Fig. 1E) and serum calcium (See Fig. 1F) levels following parathyroidectomy, in patients who were either pre or post kidney transplantation. We did not identify any statistically significant differences in the trends of PTH or calcium post parathyroidectomy, depending on whether the intervention was performed in patients who had previously received a kidney transplant versus those who had not.

Finally, we performed univariate and multivariate regression analysis to further explore factors which may impact serum PTH levels at 1 year post surgical intervention (See Table 4). Undergoing parathyroid surgery between 1999–2008 was the only factor on univariate analysis that significantly predicted a reduction in serum PTH at 1 year (*P* = 0.044).

**Table 4:** Univariate and multivariate linear regression analysis; examining the role of various clinical parameters on recurrent hyperparathyroidism post parathyroidectomy. Recurrence was defined as any patient who underwent diagnostic imaging to further investigate a rise in serum PTH. *PXT 1999–2008*; this represents the cohort managed prior to the widespread adoption of cincacalcet as a therapeutic option in this context. *PXT parathyroidectomy, SPTX subtotal parathyroidectomy*

Recurrent Hyperparathyroidism (*N* = 30)	*N*	Univariate Coefficient	SE	*P*-Value	Coefficient	Multivariate SE	*P*-Value
Male	77/155	−0.05	0.06	0.940	−0.04	0.08	0.663
Age ≥ 55	56/155	0.02	0.06	0.787	0.02	0.09	0.828
Transplanted before PTX	107/155	0.02	0.06	0.761	0.03	0.09	0.750
SPTX	108/155	0.02	0.07	0.857	0.03	0.09	0.720
PTX 1999–2008	42/155	−0.15	0.07	**0.044**	−0.14	0.11	0.207
Pre-op PTH > 600	89/142	−0.01	0.07	0.981	0.04	0.09	0.682
Pre-op PTH > 800	81/142	0.02	0.07	0.764	0.08	0.09	0.351
Pre-op PTH > 1000	72/142	0.08	0.07	0.268	0.14	0.08	0.097
Pre-op ALP > 200	58/122	−0.08	0.07	0.276	−0.11	0.08	0.187

### Evaluating factors predictive of significant post-operative hypocalcaemia (Hungry bone syndrome)

We performed univariate and multivariate regression analysis to further explore pre-operative factors predictive of HBS in this context (See Table 5). Pre-operative Alkaline phosphatase > 200 IU/L was predictive of HBS on univariate (*P* = 0.003) and multivariate (*P* = 0.002) analysis, whilst a PTH > 1000 pg/ml (*P* = 0.012) was also predictive of HBS on univariate analysis.

**Table 5:** Univariate and multivariate linear regression analysis; examining the role of various clinical parameters on developing *‘hungry bone syndrome’* post parathyroidectomy. *‘Hungry bone syndrome’* was defined as any patient who required IV calcium supplementation or developed symptomatic hypocalcaemia post parathyroidectomy. *PXT 1999–2008*; this represents the cohort managed prior to the widespread adoption of cincacalcet as a therapeutic option in this context. *PXT parathyroidectomy, SPTX subtotal parathyroidectomy, ALP Alkaline Phosphatase*

*Hungry Bone Syndrome (N* = *43)*	*N*	Univariate Coefficient	SE	*P*-Value	Coefficient	Multivariate SE	*P*-Value
Male	77/155	−0.34	0.07	0.634	0.06	0.09	0.430
Age ≥ 55	56/155	−0.26	0.07	0.728	−0.04	0.09	0.624
Transplanted before PTX	107/155	−0.67	0.08	0.367	0.08	0.09	0.361
SPTX	108/155	0.116	0.08	0.139	0.23	0.08	0.170
PTX 1999–2008	42/155	−0.225	0.08	**0.005**	−0.28	0.11	**0.012**
Pre-op PTH > 600	89/142	0.15	0.07	0.068	0.09	0.08	0.327
Pre-op PTH > 800	81/142	0.12	0.08	0.117	0.02	0.09	0.789
Pre-op PTH > 1000	72/142	0.19	0.07	**0.012**	0.13	0.08	0.126
Pre-op ALP > 200 (IU/L)	58/122	0.24	0.08	**0.003**	0.26	0.08	**0.002**

Meanwhile, undergoing a parathyroidectomy in the pre-cinacalcet era between 1999–2008 was protective against HBS on univariate (*P* = 0.005) and multivariate (*P* = 0.012) analysis.

### Changes in practice following the introduction of cinacalcet

Data from our regression models suggest that patients undergoing surgery in the pre-cinacalcet era (1999–2008), were less likely to develop recurrent HPT or HBS. In light of this we wanted to take a closer look at this cohort (See Table 6). Firstly we can see there was a significant difference in the number of patients treated with cinacalcet in advance of undergoing parathyroidectomy in the 2009–2023 cohort compared to the 1999–2008 group (93.8% Vs 9.5%, *P* < 0.005). Secondly, the timing of referral differed in that the mean pre-operative PTH in the 1999–2008 cohort was 891 compared to 1258 following the introduction of cinacalcet during the 2009–2023 era. In keeping with this, fewer patients were symptomatic pre-operatively in the 1999–2008 group (*P* = 0.032).

**Table 6:** A comparison of the pre-operative characteristics of patients who underwent surgical intervention before (1999–2008), and after (2009–2024) the widespread adoption of cincacalcet as a therapeutic option in the management of patients with renal hyperparathyroidism. Continuous variables are mean ± standard deviation, categorical variables are n (%). *PTX parathyroidectomy, SPTX subtotal parathyroidectomy*

	PTX 1999–2008 (*N* = 42)	PTX 2009–2024 (*N* = 113)	*P* value
Male	50.0% (21/42)	46.9% (53/113)	0.731
Age ≥ 55	35.7% (15/42)	35.4% (40/113)	0.971
Transplanted Pre PTX	61.9% (26/42)	65.5% (74/113)	0.828
SPTX	61.9%(26/42)	74.3% (84/113)	0.177
Mean PTH Pre PTX	891 ± 870	1258 ± 931	**0.047**
Symptomatic Pre PTX	15.0% (6/40)	32.7% (37/113)	**0.032**
Cinacalcet Pre PTX	9.5% (4/42)	93.8% (46/49)	** < 0.005**

## Discussion

The significant reduction in PTH levels following parathyroidectomy, as compared to cinacalcet treatment, emphasises the long-established role of surgical intervention in controlling severe cases of renal HPT. The data show that at one year, the mean decrease in PTH following surgery was considerably greater than in patients treated with cinacalcet, suggesting that parathyroidectomy remains a very effective option for achieving rapid and substantial reductions in PTH levels. However, it is also noteworthy that cinacalcet was associated with reasonable PTH control, and remains an effective alternative particularly in patients who may not be immediate candidates for surgery or for whom the surgical risks are significant.

The indications for surgical parathyroidectomy in the context of renal HPT, as suggested by international guidelines, remain inconclusive largely owing to the absence of high quality evidence. The National Kidney Foundation-Dialysis Outcomes Quality Initiative (NKF-DOQI) suggest parathyroidectomy should be recommended in patients with severe HPT, with persistent serum levels of PTH > 800 pg/mL, with associated hypercalcemia and/or hyperphosphatemia that is refractory to medical therapy [12]. The Japanese Society for Dialysis Therapy recommend parathyroidectomy for severe renal HPT, where PTH > 500 pg/mL following appropriate medical management [11]. Finally, the Kidney Disease Improving Global Outcomes (KDIGO) guidelines recommend surgical parathyroidectomy in patients with CKD stages 3–5 with severe HPT who fail to respond to medical/pharmacological therapy [10]. The KDIGO guidelines do not recommend a PTH level that should prompt consideration of surgical referral. Our data reflects a national trend whereby patients with renal HPT were generally commenced on cinacalcet when serum PTH levels reached 800pg/ml, and surgical referral was opted for once the PTH level had risen to between 1000–1200 pg/ml despite appropriate medical intervention. Of course though these were general trends observed nationally, our data does not conclusively capture isolated incidences where surgery may have been considered for differing reasons on a case by case basis. A key objective of our review was to evaluate the validity of this general approach and identify whether or not it could be optimised.

From our data we can see, the 2 most commonly encountered complications of surgery were recurrent HPT and HBS. Previous studies have reported recurrence rates between 3.7–20% post 3.5 gland SPTX and between 2.2–12% post TPTX + AT [16–21]. The criteria for documenting recurrent disease following parathyroid surgery, varied amongst previous studies, leading to considerable differences in reported outcomes. Overall we found that although 30 patients (19.3%) were investigated post-operatively for the presence of a potential recurrence, only 4 patients (2.6%) required further surgery to manage recurrent HPT. This compares favourably with data published from other centres [16, 17]. Interestingly when we looked at factors predictive of potential recurrence, patients who had a documented serum PTH level > 1000 pg/ml, were found to have significantly higher PTH levels at 5 years. Similarly results from our regression model found that undergoing surgery in the pre-cinacalcet era (1999–2008) was protective against recurrent HPT. Furthermore, we note that the mean pre-operative PTH was significantly lower during this period (891 ± 870 Vs 1258 ± 931). Our findings suggest that early intervention when PTH levels < 1000 pg/ml could potentially reduce rates of recurrent HPT. This finding underscores the importance of timely surgical referral to mitigate long-term risks associated with persistent HPT.

HBS was first described in 1948 in patients with prolonged hypocalcaemia post parathyroidectomy for primary HPT [22]. Previous studies report incidence rates varying between 27.4% and 51.2% post surgical management of renal HPT [23–26]. HBS, was observed in 27.7% of our cohort. This complication, while well-documented, poses a significant management challenge due to the potential for prolonged hospitalisation and intensive post-operative care. Pre-operative alkaline phosphatase levels above 200 IU/L were a strong predictor of HBS, aligning with existing knowledge that elevated markers of bone turnover increase the risk of this complication [23, 27, 28]. Interestingly we also found that patients with pre-operative PTH levels > 1000 pg/ml were more likely to develop HBS, this further emphasises the long term patient benefits of early surgical referral.

Previous studies have focused on the approach and timing of surgery in the context of renal HPT. A recent study reviewed the American College of Surgeons NSQIP data from 2005 to 2013, which included a cohort of 1,130 patients; 68% of whom had undergone a SPTX versus 32% of whom had undergone a TPTX-AT. The only significant finding was that patients undergoing TPTX-AT had a longer hospital stay. There were no differences in complication rates or 30-day readmission rates [29]. Similarly Chen et al. published a meta-analysis of 13 comparative studies, including 1,685 patients, and concluded there was no significant difference between the 2 approaches with regard to initial success, recurrence, reoperation rate, or long-term hypocalcaemia [13]. This equivalency has been further outlined in a more recent meta-analysis published this year by Albuck et al. [14]. Our analysis found no difference in terms of recurrence, or incidence of HBS according to surgical approach, with both cohorts also demonstrating similar outcomes with regards to mean PTH level at 3 and 5 years. Previous evidence also suggested parathyroidectomy post kidney transplantation may incur temporary allograft dysfunction [30–32]. Across our cohort there were only 2 incidences of temporary graft dysfunction (1.9%), whilst rates of recurrence and HBS were similar regardless of the timing of intervention (pre/post kidney transplantation).

While this study provides valuable insights, it is not without limitations. The retrospective nature of the analysis, while allowing for a broad assessment of long-term trends, may introduce biases related to data collection and patient selection. The near-universal use of cinacalcet in patients referred for surgery after 2009 (93.8% vs. 9.5% in the earlier cohort) suggests that calcimimetics are being used not only as an alternative but also as a preparatory measure before surgical intervention. While this approach allows for the stabilization of PTH levels and potentially improved pre-operative conditions, the long-term impact of this practice on surgical outcomes and recurrence rates warrants further investigation. Future studies should explore the optimal timing for transitioning patients from medical to surgical management to minimize the risks of delayed intervention.

In conclusion, this study underscores the evolving practice of managing renal hyperparathyroidism in the era of calcimimetics, highlighting the ongoing need for individualized treatment strategies that balance medical and surgical approaches to optimize patient outcomes.

## Supplementary Information

Below is the link to the electronic supplementary material.

Supplementary file1 (DOCX 15 KB)

## Data Availability

Original data may be made available upon reasonable request.
