# Supplementary material for: A retrospective analysis of the management of renal hyperparathyroidism; evaluating changes in practice and outcome in an era of calcimimetics
Source: Langenbecks Arch Surg. 2025 Jun 2;410(1):172. doi: 10.1007/s00423-025-03744-2 (PMC12129844; doi:10.1007/s00423-025-03744-2)
Supplement: Supplementary file 1 — Supplementary file1 (DOCX 15 KB) [file 423_2025_3744_MOESM1_ESM.docx]

|  | **Surgical Cohort** | **Cinacalcet Cohort** |
| --- | --- | --- |
| Beaumont Hospital | 68 | 203 |
| Galway University Hospital | 22 | - |
| Tallaght University Hospital | 41 | - |
| Cork University Hospital | 24 | - |
| **Total** | **155** | **203** |

**Supplementary Table 1.**  A breakdown of the centres involved in the study and the specific

numbers provided by each.
